# HPLC Quantification of Thymoquinone Extracted from *Nigella sativa* L. (*Ranunculaceae*) Seeds and Antibacterial Activity of Its Extracts against *Bacillus* Species

**DOI:** 10.1155/2021/6645680

**Published:** 2021-04-20

**Authors:** Nida Habib, Shahnaz Choudhry

**Affiliations:** Department of Biotechnology, Kinnaird College for Women, 93-Jail Road, Lahore, Pakistan

## Abstract

The medicinal importance of *Nigella sativa* seeds for treating various ailments is portrayed by its traditional uses. Owing to its immense pharmacological importance, the thymoquinone phytoconstituent of *N. sativa* can prove beneficial for the South Asian countries including Pakistan, where this seed is commonly produced and healthcare facilities are limited. In this study, the antibacterial activity of various extracts of *N. sativa* seeds, extracted thymoquinone, and oil samples have been investigated against *Bacillus subtilis* and *Bacillus licheniformis* using well and disc diffusion assay. The inhibition zones ranged between 7 and 44 mm against both the bacterial strains by well diffusion assay, while disc diffusion assay provided inhibition zones in the range of 7–23 mm. Commercial and local Kalonji oil samples were included in the study. Oil samples dissolved in methanol showed increased inhibition of bacteria. However, the extracted thymoquinone showed highest antibacterial activity. Medicine formulated using thymoquinone will prove to be an herbal alternate against the resistant microbiota associated with bacterial infections. Antibacterial activity against some *Bacillus* species will help signify the effect on normal gut flora when oral therapy is followed. Trying different extraction protocols can help increase extraction efficiency. Study on extraction of thymoquinone in local produce of black seed can be fruitful for conducting the stability studies and can help to gain maximum benefits from the bioactives. The crude extracts from 10 g of these seeds were subjected to preliminary phytochemical investigation. Results showed that although methanol extract had the presence of maximum phytochemicals, hexane extract was the most potent in terms of antibacterial activity. Thymoquinone, a therapeutically important bioactive in *N. sativa* seed, was extracted employing both solvents. TLC assay and UV spectroscopy were used for its qualitative assessment, while HPLC-UV quantification showed that 250 mg/mL of methanol extract had 368.3 *μ*g/mL thymoquinone, while its successive extraction yielded 32.94 *μ*g/mL thymoquinone.

## 1. Introduction

Plants are source of phytoconstituents that act as contemporary drugs which are also a part of modern therapeutics [1]. 25–30% compounds in medical prescriptions in developed countries are from the plant origin. Drugs obtained from plant sources are contributing 30,000 million USD to the world market of drugs [2]. According to the World Health Organization, in developing countries, this percentage is 80% [3]. Three-fourth of the population in the underdeveloped countries rely upon plant-based medicines for primary healthcare needs. One of the reasons for this situation is the high cost of allopathic medicines [4]. Bioactive constituents act as antioxidant [5], antidiabetic [6], anti-inflammatory, antibacterial, and antifungal [7] agents.

**Figure 1:**
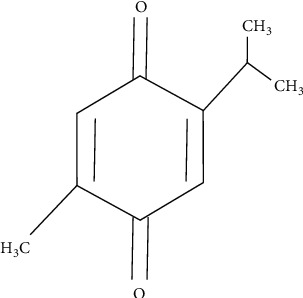
Chemical structure of thymoquinone (an oxygenated monoterpene).

The term “phytochemicals” refers to compounds made by plants that can affect human health. *Nigella sativa* is a plant that is cultivated as a crop for its seeds [8]. These seeds are commonly referred to as black seeds in East African, Middle Eastern, Mediterranean, and South Asian countries. The seed is important for centuries owing to its culinary and medicinal efficacy [9, 10]. *N. sativa* seed seems to possess a range of medically advantageous substances that can be employed for curing human ailments and other major diseases with negligible chances of toxicity, if any [11]. *N. sativa* seeds are also known as black cumin and Kalonji. *N. sativa* is a promising plant source of bioactive constituents such as thymoquinone (TQ), *α*-pinene, *p*-cymene, and monoterpenes [12, 13]. Thymoquinone in the *N. sativa* seed is known to help protect against liver damage and is commonly mentioned as hepatoprotective mainly being active against fibrosis [14]. Thymoquinone has also been identified as the phytochemical largely responsible for the medicinal properties of the black seed [15]. Chemical structure of thymoquinone is given in Figure 1. *N. sativa* seed oil, its methanolic extract, volatile oil, and the thymoquinone constituent have been tested for their therapeutic role using rat models [16].

Thus, thymoquinone is held in great esteem for imparting the antibacterial characteristic to the divine black seed. Moreover, it can be used as an antibiofilm in the form of a bioactive and is a prominent highlight in treatment of infection [17]. Despite the long historical use of black seed in different health-related issues, human studies for the use of thymoquinone are not as much accelerated [18]. Thus, the clinical and pharmacokinetic studies are required to encourage the use of thymoquinone. Clinical trials are ready to enter next phase as no significant toxicity was observed in the preliminary trials [19].

Thymoquinone from *N. sativa* can prove beneficial for the South Asian countries including Pakistan where the black seed is commonly produced and healthcare facilities are limited. The medicine formulated using thymoquinone will prove to be herbal alternate against bacterial infections [20]. Antibacterial activity against some *Bacillus* species can help signify the effect on normal gut flora when oral therapy is followed. Trying different extraction protocols can help increase extraction efficiency [21]. The study on presence, extraction, and purification of thymoquinone in the local produce of black seed can be fruitful for conducting the stability studies and can help to gain maximum benefits from the bioactives.

Standardization of herbal formulations in terms of quality of raw materials, manufacturing practices, and composition is important to ensure quality and optimum levels of active principles for their biopotency. No similar reports were found in the literature regarding the quantification of thymoquinone in *N. sativa* seed extracts. As most of the biological activities of black seed are due to TQ and since the seeds are the actual source of TQ [22], we undertook a comparative study of the level of TQ in the seeds and the assayed TQ in relation to antibacterial activity. This was to reinforce the pros of using the seeds as a whole in folklore therapy. The present work employed an HPLC method for the determination of thymoquinone in *N. sativa* seed extract. The method is selective for the analysis of thymoquinone with added advantages of low cost of reagents, speed and minimal sample preparation, as well as satisfactory precision and accuracy. This is a valuable research work in terms of quantification of thymoquinone in *N. sativa* seed extract using HPLC.

## 2. Materials and Methods

*N. sativa* seeds were purchased from a market in Lahore, Pakistan. All chemicals used were of analytical grade. Commercially available chloramphenicol was purchased from Remington pharmaceutical.

### 2.1. Preparation of Extracts

Kalonji seeds were powdered using mortar and pestle. The powdered material was stored in falcon tubes and covered with aluminum foil. 10 g of Kalonji seeds were extracted using 40 mL of different solvent each time. Solvents used were methanol, ethanol, diethyl ether, hexane, and water. The extraction mixture was left for one week. After one week, the extracts were filtered [23, 24].

### 2.2. Detection of Phytochemicals

Various phytochemicals such as alkaloids, terpenoids, flavonoids, glycosides, phlobatannins, and tannic acids were detected using standard protocols [25, 26]. Wagner test was performed for alkaloids. The extract was mixed with few drops of the Wagner reagent (iodine: 1.27 g; KI: 2 g in 100 mL of water) in a test tube. A red-brown precipitate confirmed a positive test. Salkowski test for terpenoids was done by mixing 2 mL of chloroform and 3 mL sulphuric acid with 2 mL of extract. Red-brown color at the interface indicated presence of terpenoids. Aluminum chloride test used 1 mL of 1% aluminum chloride solution which was added to extract, shaken, and observed for a yellow coloration. Dilute NaOH and HCl was added that turned yellow solution colorless indicating a positive result.

Tannic acids were detected by ferric chloride test in which the extract was mixed with few millilitres of 45% ethanol solution for 3 minutes and water was added. Ferric chloride drops changed the color of the solution to greenish black. Frothing test indicated the presence of saponins when extract was mixed with distilled water and shaken vigorously. Carbohydrates were detected by shaking the extract with water and adding few drops of Molisch reagent. Addition of concentrated sulphuric acid resulted in the formation of a brown ring at the interface as a positive result [11, 23].

Proteins in extracts were detected using 5 mL distilled water and 1 mL extract and left to stand for 3 hours. 2 mL of this is tested with Millon's reagent with shaking. Yellow precipitates showed presence of proteins. Glycosides test uses dilute sulphuric acid that is added to the extracts. 20% KOH solution is added. A mixture of 10 mL Fehling's solution A and B was added. Boiling of 5 minutes developed red precipitates. This indicated the presence of glycosides. Phlobatannins test uses 1 mL of extract and 1 mL of 1% HCL to give red precipitates as a positive test. Reducing sugars were detected by shaking the extract vigorously with water, and equal volumes of Fehling's solution A and B are added. Brick red precipitation is the confirmation of reducing sugars [23, 27]. Vitamin C is indicated by a test in which diphenylhydrazine dissolved in concentrated sulphuric acid was added to extract, and development of yellow color confirmed the presence of vitamin C [28].

## 3. Microbiological Assessment for Antibacterial Activity

### 3.1. Well Diffusion Assay

Agar well diffusion method was used to investigate the antibacterial activity of *N. sativa* seeds against *B. subtilis* and *B. licheniformis.* Sterilized Petri plates were poured with nutrient agar and swabbed with 24 hours bacterial culture. 6 mm deep and 6 mm wide wells were bore into agar plates and injected with extracts at 1.25 mg/*µ*L, 2.5 mg/*µ*L, and 5 mg/*µ*L concentrations. The treated Petri dishes were incubated for 24 hours at 37°C. At the end of 24 hours, the inhibition zones formed on the surface of media were measured in mm. The antibacterial activity was expressed as the mean diameter of inhibition zone in mm [29]. Chloramphenicol served as positive control, and respective solvent served as negative control.

### 3.2. Disc Diffusion Assay

Petri dishes were sterilized and poured with sterilized nutrient agar. These were then inoculated with an overnight inoculum of bacteria. Discs were made using extracts of different concentrations. 20 *µ*L was the maximum capacity of the discs. These were placed on Petri dishes, pressed firmly, and the plates were then stored at a temperature of 4°C for about 2 hours. The positive control was standard antibiotic: ciprofloxacin 5 *µ*g/disc. Solvent only served as the negative control. The plates were then incubated for 24 hours at 37°C. Inhibition zones that developed after 24 hours were measured in mm [17, 30]. The zone of inhibition in mm was used to determine the antibacterial activity of the prepared extracts [31].

### 3.3. Isolation of Thymoquinone from *N. sativa* Seeds

10 g of powdered seed was soaked in 70 mL of 80% methanol, and 4 hours of shaking was done. This was filtered and stored at 4°C. The filtered marc was again soaked in 70 mL of methanol (80%) and shaking was done for 4 hours followed by filtration. The combined filtrates were diluted to have 50% methanol concentration [32]. Solvent extraction was done using chloroform, using multiple batch extraction. 10 mL of chloroform was used each time to obtain the lower organic layer. This extract was run through a silica gel column. The silica gel was made by dissolving silica powder in distilled water to obtain slurry. The packed column was run with solvent system comprising hexane and dichloromethane (7 : 3), and fractions were collected after short intervals in test tubes. The fractions were stored in screw-capped test tubes at 4°C in order to prevent evaporation losses.

### 3.4. Thin Layer Chromatography

Qualitative analysis of the fraction obtained after column chromatography (CC) of the methanolic extract was done using thin layer chromatography (TLC) on commercially available cards [33]. A spot of the standard thymoquinone, a spot of methanol extract and spots for the two layers obtained after solvent extraction, and a spot of extract after CC were analyzed. TLC cards were used for thin layer chromatography. Samples were applied as spots, using Pasteur pipette. The TLC card was placed in a beaker to obtain an ascending thin layer chromatogram.

The solvent system used was hexane and dichloromethane (1 : 1). The card was allowed to run till the solvent had travelled 3/4 of the card [34]. The cards were then removed from the solvent, and the mobile phase was marked. The cards were allowed to dry for visualization.

The chromatogram obtained was viewed under UV lamp (200–300 nm), and visualized spots were marked for calculating *R*_*f*_ values. The *R*_*f*_ value is interpreted as the distance that is moved by the compound from the point of origin divided by the distance the solvent has moved from the starting point.

### 3.5. UV-Vis Spectroscopy

The UV-Vis spectroscope is equipped with deuterium lamp emitting ultraviolet radiation (200–300 nm). Determination of UV *λ*_max_ helps identification of molecule [31]. Absorbance was taken at 265, 260, 253, and 250 nm to detect the presence of thymoquinone. The absorbance was measured in triplicate at each wavelength.

### 3.6. High-Performance Liquid Chromatography

A simple, sensitive, and precise reversed phase high-performance liquid chromatography (RP-HPLC) method was used for quantitative determination of thymoquinone from the methanolic extracts of *N. sativa* seeds [35].

### 3.7. Reagents and Instrumentation

Authentic standard of thymoquinone was purchased from Sigma-Aldrich, USA. HPLC grade methanol and water were purchased from Merck. HPLC analysis was carried out using a Hitachi LaChrom Elite liquid chromatograph that was equipped with an autosampler Model L-200, degasser Model L-2130 pump, and UV Model L-2400 (Tokyo, Japan). The analysis was carried out using C18 (150 mm × 4.6 mm) column packed with 5 *μ*m Interstil ODS-3v particles. Data acquisition was done using EZChrom Elite software.

### 3.8. Stock Solution and HPLC Conditions

Accurately weighed 10 mg of standard thymoquinone (purity 99%) was dissolved in 10 ml methanol to give a concentration of 1000 *µ*g/mL. This solution was used as stock solution for thymoquinone. RP-HPLC analysis was carried out with water and methanol (40 : 60, v/v) as the mobile phase in an isocratic system with a flow rate of 1.5 mL/min and 254 nm as detection wavelength [36]. The mobile phase and samples were filtered through 0.22 *μ*m Dura PVDF membrane aqueous filter, Millex GV, filter unit, and degassed under vacuum prior to injection into the instrument.

Under the optimized chromatographic condition, 10 *μ*L of sample solution was injected into the system. The identities of peaks of thymoquinone were determined by comparing the chromatogram of each sample solution with that of standard thymoquinone. The amount of thymoquinone present in the sample was calculated.

Chromatogram of standard thymoquinone and that of the sample were obtained. Thymoquinone was detected at retention time between 6^th^ and 7^th^ minute in a 10-minute cycle. The difference between retention time of standard thymoquinone and that detected in methanol sample was 0.27% (i.e. < 5%). The difference between retention time of standard thymoquinone and that of isolated thymoquinone was 1.2% (i.e. < 5%). Concentration of thymoquinone in standard solution (SND) was 1 mg/ml, while percentage concentration of *N. sativa* seed powder in methanol extract (SMP) was 250 mg/mL. So, by using the following formulas, we calculated the concentration of thymoquinone in the prepared extract:

(1) $$\begin{array}{c} \frac{\text{peak area of SMP}}{\text{peak area of SND}}\times \frac{\text{concentration of SND}}{\text{concentration of SMP}}\times 100, \end{array}$$

(2) $$\begin{array}{c} \text{concentration of sample}=\frac{\text{peak area of sample}}{\text{response factor}}. \end{array}$$

## 4. Results and Discussion

### 4.1. Phytochemical Analysis

The phytochemicals were detected in *N. sativa* seeds using standard protocol and are presented in Table 1. The results show that *N. sativa* seed contains no proteins or reducing sugars. All other phytochemicals were variably detected in different extracts. Alkaloids, glycosides, terpenoids, flavonoids, tannins, and many more bioactive constituents are found in plants [37, 38]. Order of maximum phytochemicals in different extracts is as follows:

(3) $$\begin{array}{c} \text{methanol}>\text{ethanol}=\text{hexane}=\text{ether}>\text{aqueous}. \end{array}$$

**Figure 2:**
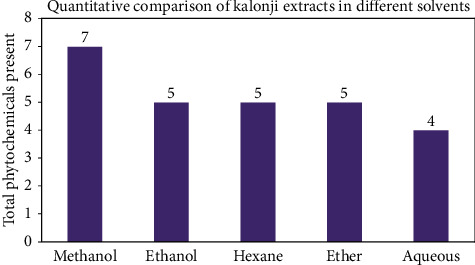
Qualitative comparison of Kalonji extracts prepared in different solvents.

Methanol extract showed maximum number of phytochemicals, whereas aqueous extract was poor in extraction efficiency. Quantitative comparison of Kalonji extracts made in different solvents is shown in Figure 2.

### 4.2. Antibacterial Activity

Antimicrobials are the base of clinical medicine. Antimicrobial resistance in pathogenic microorganisms became prevalent in the late 20^th^ century [39, 40]. The rate of microbial infections is increasing along with antimicrobial resistance. This has raised demand for a need to discover novel solutions using natural plant products [41, 42]. *N. sativa* seeds display strong antibacterial properties with thymoquinone as major phytochemical involved.

The antibacterial activity has been assessed quantitatively as either presence or absence of the inhibition zone. The present study employed both agar well diffusion and disc diffusion assays. The inhibition zones obtained after agar well diffusion and disc diffusion assay are given in Tables 2 and 3, respectively. Agar well diffusion method proved to be more efficient in aiding extract diffusion. Hence, we will prefer the more reliable (well diffusion assay) results. The results suggested that both strains were susceptible to antibacterial phytoconstituents of seed extracts. Thymoquinone obtained from seeds of *N. sativa* shows broad spectrum activities against strains of Gram-positive and Gram-negative bacteria including *Bacillus* and also inhibits bacterial biofilm formation [43]. The ascending order of antibacterial activity by extracts made in different solvents is as follows:

(4) $$\begin{array}{c} \text{hexane}>\text{methanol}>\text{ethanol}>\text{diethyl ether}>\text{aqueous}. \end{array}$$

**Figure 3:**
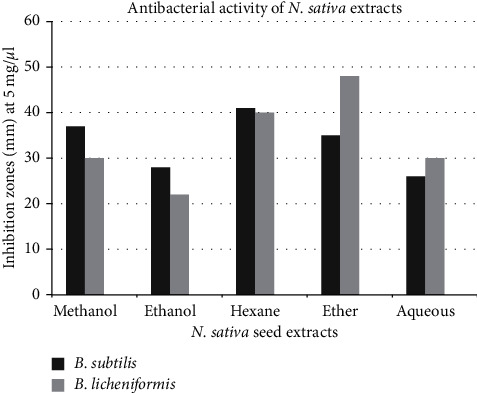
Antibacterial activity of *N. sativa* seed extracts against Gram-positive *Bacillus* strains.

The antibacterial activity displayed by Kalonji extracts is given in Figure 3. Extraction of botanical compounds including antibacterial compounds is dependent on the type of solvent used. Organic solvents like methanol improve extraction efficiency and, therefore, improve antibacterial activity as compared to water extracts [44]. The methanolic extract of the seed also displays a larger inhibition zone on Gram-positive in comparison with Gram-negative bacteria [45]. Aqueous extract of *N. sativa* seeds shows lesser antibacterial activity due to less availability of phytochemicals (antibacterial agents) in this extract as all extracts except aqueous extract have oily droplets [46]. Antibacterial activity of oil samples is given in Table 4.

Monoterpenes are among the many bioactive components of *N. sativa* seed, and their mode of antimicrobial action is related to their ability to inactivate microbial adhesion, enzymes, and cell envelope proteins [47]. Terpenoids, alkaloids, and saponins are majorly involved in membrane disruption [48]. The antibacterial effect of the plant extract justifies its use in traditional medicine. The ascending order of antibacterial activity of all test samples is as follows:

(5) $$\begin{array}{c} \text{extracted TQ}>\text{hexane}>\text{methanol}>\text{local oil}>\text{commercial oil.} \end{array}$$

Increased resistance of bacteria against current antibiotics and production of synthetic drugs by pharmaceutical industry results in high cost for consumer posing a need for safe alternatives. Medicinal plants provide a more reliable and economical alternative against many bacterial ailments. Findings of the present study demonstrate that seeds of *N. sativa* are blessed with numerous phytoconstituents that impart medicinal virtues to the seeds including antibacterial activity.

Both strains of bacteria were found to be equally susceptible to the antibacterial activity of the extracts. However, standard antibiotic was stronger than extracts. The susceptibility pattern of standard antibiotics against *Bacillus* strains used in this study is provided in Table 5. Antibacterial activity of *N. sativa* seed extracts increased with increasing the concentration of extracts. Chloroform extracts showed negligible antibacterial activity and hence are not recorded. The plant extract and its fraction were not very effective at inhibiting the nonpathogenic bacterial strains, which indicates that use of the plant extract as medicine will only affect pathogenic bacteria and not the normal flora of the gut cavity [49]. Previously, an inhibition zone of 23 mm was the highest value recorded for ethanolic extract at 150 mg/mL [46]. The ethanolic extract in this study gave 28 mm zone, and methanolic extract gave 37 mm zone at just 5 mg/*µ*L.

The aqueous extracts have been reported to be more effective against Gram-positive *B. subtilis* bacteria as compared to ethanolic extracts [46], but it was opposite in our study when the extracts were tested on *B. licheniformis,* so it cannot be said that aqueous extracts are always better at inhibiting Gram-positive bacteria [50]. Thymoquinone column fraction displayed mild to moderate activity. Similar activity has been reported previously by extracted bioactive compounds [35]. Absence of inhibition zone does not mean absence of active compound as inhibition zone values improved on increasing the concentration of the extracts used. Also, fractionation of thymoquinone gave the highest inhibition zone indicating that its high concentration plays a role in the antibacterial action.

Methanol and hexane extracts were strongly positive for terpenoids in phytochemical analysis and showed considerable antibacterial activity; therefore, antibacterial property can be linked to presence of terpenoids [51] and subsequently to thymoquinone which is a monoterpene, and so its levels were looked for in methanol extract as well as in column fraction obtained by a protocol that employs both these solvents. Thus, both polar and nonpolar solvents were employed in the extraction process to achieve maximum extraction efficiency.

### 4.3. Thymoquinone Analysis

The methanolic extract was subjected to solvent extraction with chloroform in a separating funnel. Exhaustive extraction (EE) is carried out with different solvents of increasing polarity in order to extract as much as possible the most active components with highest biological activity. This resulted in the extract being divided into two distinct layers. Both the layers were separated. The lower organic layer having milky white appearance was allowed to run through a silica column giving colorless fractions, each collected after a minute of interval. The solvent system used was hexane and dichloromethane (7 : 3).

### 4.4. Thin Layer Chromatography

**Figure 4:**
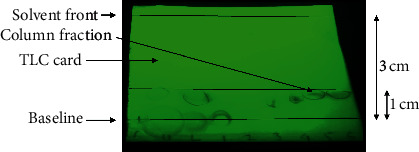
TLC card for methanolic extract of Kalonji seeds for the extraction of thymoquinone viewed under UV lamp (extract, upper layer, lower layer, and fractions) as seen under the UV lamp.

**Figure 5:**
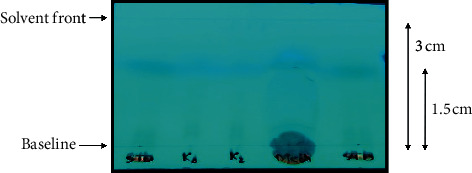
TLC card under the UV lamp showing standard thymoquinone run on both sides against column fractions (K1 and K2) and methanol extract of Kalonji seeds.

The original 80% methanolic extract, the upper and the lower organic layers obtained by solvent extraction, and 6 of randomly selected colorless fractions that were obtained by column chromatography of the methanolic extract were subjected to TLC. TLC was performed using hexane and dichloromethane (1 : 1) as the mobile phase. The spots obtained under the UV lamp were marked (Figures 4 and 5). The *R*_*f*_ value of the fractions was found to be 0.3.

### 4.5. Determination of UV *λ*_max_

The absorbance of fractions was measured in the UV range using a T80 UV-Vis spectrophotometer, PG Instruments Ltd., UK. The wavelengths selected in the UV range were 265, 253, and 250 nm (Table 6). The absorbance was recorded in triplicate with hexane and dichloromethane (7 : 3) as blank. The UV *λ*_max_ was found to be 253 nm each time and is tabulated in Table 6.

### 4.6. Quantification of Thymoquinone

**Figure 6:**
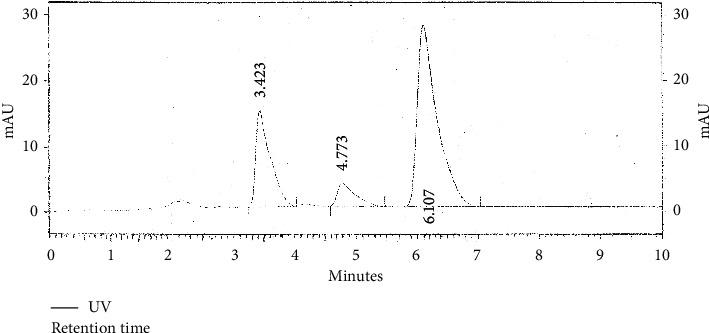
HPLC chromatogram of standard thymoquinone solution.

**Figure 7:**
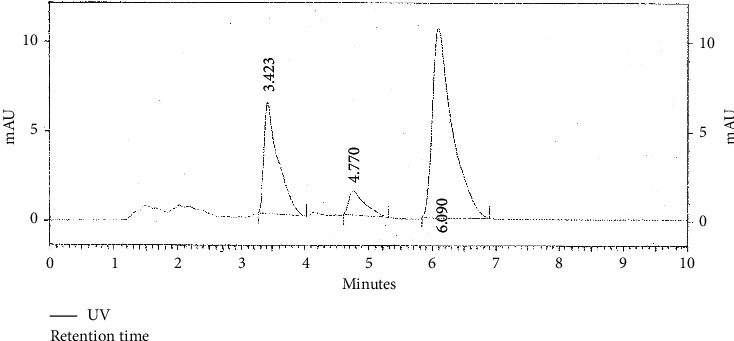
HPLC chromatogram of methanol extract of *N. sativa* seeds.

**Figure 8:**
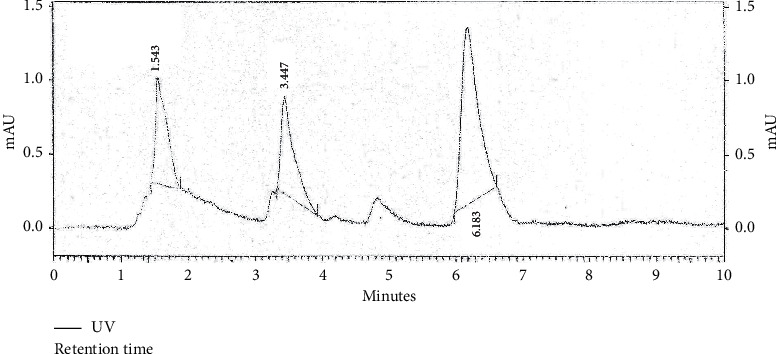
HPLC chromatogram of thymoquinone isolated from *N. sativa* seeds.

HPLC chromatograms for standard thymoquinone, methanolic Kalonji seed extract, and isolated thymoquinone were obtained and are shown in Figures 6–8.

Thymoquinone is well known as the major bioactive found in *N. sativa* seeds. Previously, reported levels of thymoquinone are 5 *µ*g/mL or less [52]. The amount of thymoquinone in methanol sample using the HPLC-UV method was found to be 368.3 *µ*g/mL and 32.94 *µ*g/mL in isolated thymoquinone. These are impressive quantities as compared to the existing data. This can be attributed to the care taken while preparing, storing, and analyzing samples that were kept in reagent bottles covered with aluminum foil and refrigerated after each use. This extraction detection method used sample that was syringe filtered prior to reverse phase HPLC analysis using methanol-water gradient, and TQ extraction was enhanced by purification using solid phase extraction both of which proved effective in resolving TQ peak. This minimized the interference from other irrelevant peaks.

Most of the biological activities of black seed are due to thymoquinone, and it has definite physiological action on human body. Traditional source of thymoquinone is use of whole black seed, while its making into a pharmacological drug or a nutraceutical requires extraction and purification to obtain concentrated bioactive is a comparatively expensive way to reap the benefits. The comparative study carried out to study TQ levels in crude extracts and extract made by isolation protocol proved the point. For this, HPLC as an analytical method was employed that minimizes interference from other constituents [36]. This method was simple and rapid, and preparation of the mobile phase was very easy. Microbiological methods are time-consuming, and so HPLC proved as an efficient alternative [52].

Previously, 5.19 ± 0.43 *µ*g/mL thymoquinone was reported in methanolic extract, whereas in present study, the amount is far high. Quantification of thymoquinone from *N. sativa* whole seed has not been done using HPLC. Thus, ingesting *N. sativa* whole seed still proves to be significantly useful as simple methanolic extraction and subsequent thymoquinone extraction from *N. sativa* whole seed give evidence of high amount of bioactive in them rather than purchase of high-priced oil in comparison with seed. The calculated percentage of TQ in the seed extract was 1.5%, higher than 1% recorded in previous studies [32]. This amount was considerably higher than assayed TQ. Oil although having potency of the bioactive as an antibacterial agent was behind the seed extracts. TQ fraction with greater antibacterial activity is still not very impressive as the fatigue required to obtain it. As a result of this study, methanol proved to be the most efficient solvent for phytochemical extraction. Hexane and methanol extracts were more potent in terms of antibacterial activity. The black seed and its products have negligible toxicity [53, 54].

## 5. Conclusion

*N. sativa* L. seeds were used for qualitative and quantitative analysis of thymoquinone, an important biologically active compound. The methanolic extract and the isolated thymoquinone from 10 g of seeds were effective against *B. subtilis* and *B. licheniformis*. This was due to the significant TQ content present in *N. sativa* seeds. This shows that these seeds have bactericidal properties with the advantage of preventing elimination of healthy bacteria in the gut. In this study, well diffusion method appears to be more effective than disc diffusion method as indicated by inhibition zone values. HPLC analysis resulted in 368.3 *μ*g/mL of thymoquinone in the methanolic extract, and as an isolated compound, it was quantified to be 32.94 *μ*g/mL. This proved to be simple and efficient method for thymoquinone extraction from *N. sativa* L. seeds. Thymoquinone can be added in fortified functional foods or in nutraceutical. Further clinical research is required to boost its use as pharmaceutical preparation.

## Figures and Tables

**Table 1:** Qualitative analysis of phytoconstituents in the *N. sativa* seed extracts.

Sr. no.	Phytochemicals	Methanol extract	Ethanol extract	Hexane extract	Ether extract	Aqueous extract
1	Alkaloids	++	−	+	++	−
2	Carbohydrates	−	+	−	+	−
3	Flavonoids	++	++	−	++	+
4	Glycosides	++	+	−	−	−
5	Phlobatannins	−	−	−	−	−
6	Proteins	−	−	−	−	−
7	Reducing sugars	−	−	−	−	−
8	Saponins	++	−	+++	−	+
9	Tannins	+	−	+	−	+
10	Terpenoids	+++	+	+++	+	−
11	Vitamin C	+++	+++	+++	+++	+++

^+^Presence of phytochemicals. ^−^Absence of phytochemicals. ^++^Moderate concentration. ^+++^High concentration.

**Table 2:** Antibacterial activity of *N. sativa* extracts using well diffusion assay.

Tested bacteria	Concentration (mg/*µ*L)	Methanol extract	Ethanol extract	Hexane extract	Ether extract	Aqueous extract
	Inhibition zones (mm)					
*B. subtilis*	1.25	9	8	11	N.I.*∗*	8
	2.5	21	13	17	19	13
	5	37	28	41	35	26

*B. licheniformis*	1.25	7	7	N.I.	N.I.	N.I.
	2.5	26	21	19	10	11
	5	30	22	40	40	30

N.I.*∗*: no inhibition.

**Table 3:** Antibacterial activity of *N. sativa* extracts using disc diffusion assay.

Tested bacteria	Concentration (mg/*µ*l)	Methanol extract	Ethanol extract	Hexane extract	Ether extract	Aqueous Extract
	Inhibition zones (mm)					
*B. subtilis*	1.25	8	7	N.I.*∗*	8	N.I.
	2.5	9	5	9	N.I.	N.I.
	5	N.I.	23	19	12	N.I.

*B. licheniformis*	1.25	N.I.	N.I.	N.I.	N.I.	N.I.
	2.5	N.I.	7	N.I.	5	N.I.
	5	11	N.I.	N.I.	N.I.	9

N.I.*∗*: no inhibition.

**Table 4:** Antimicrobial activity of the oil samples.

Tested bacteria	Kalonji oil (commercial)	Commercial oil in methanol (5 mg/*µ*L)	Kalonji oil local vendor	Local oil in methanol (5 mg/*µ*L)	Isolated thymoquinone
	Inhibition zones (mm)				
*B. subtilis*	25	32	30	31	44
*B. licheniformis*	21	22	19	20	38

**Table 5:** Antimicrobial susceptibility pattern of standard antibiotics against selected *Bacillus* species.

*Bacterial isolate*	*Ciprofloxacin (CPX)*	*Chloramphenicol (CHL)*	

*B. subtilis*	S*∗*	S	
*B. licheniformis*	S	S	

*Antibiotics*	*Susceptible (S)*	*Intermediate*	*Resistant*

Ciprofloxacin (CPX)	≥22	17–21	≤16
Chloramphenicol (CHL)	≥24	21–23	≤20

S*∗*: susceptible.

**Table 6:** The absorbance of the fractions obtained by column chromatography of 80% methanolic extract in the UV spectra.

*ƛ* (nm)	Absorbance		
	265	253	250
1	−0.039	0.099	−0.076
2	0.139	0.154	−0.386
3	−0.039	0.098	−0.033

## Data Availability

The data used to support the findings of this study are included within the article.
